# Ricin uses arginine 235 as an anchor residue to bind to P-proteins of the ribosomal stalk

**DOI:** 10.1038/srep42912

**Published:** 2017-02-23

**Authors:** Yijun Zhou, Xiao-Ping Li, Brian Y. Chen, Nilgun E. Tumer

**Affiliations:** 1 Department of Plant Biology and Pathology, School of Environmental and Biological Sciences, Rutgers University, New Brunswick, New Jersey 08901-8520, USA; 2 Department of Computer Science and Engineering, Lehigh University, Bethlehem, Pennsylvania 18015-3084, USA

## Abstract

Ricin toxin A chain (RTA) binds to stalk P-proteins to reach the α–sarcin/ricin loop (SRL) where it cleaves a conserved adenine. Arginine residues at the RTA/RTB interface are involved in this interaction. To investigate the individual contribution of each arginine, we generated single, double and triple arginine mutations in RTA. The R235A mutation reduced toxicity and depurination activity more than any other single arginine mutation in yeast. Further reduction in toxicity, depurination activity and ribosome binding was observed when R235A was combined with a mutation in a nearby arginine. RTA interacts with the ribosome via a two-step process, which involves slow and fast interactions. Single arginine mutations eliminated the fast interactions with the ribosome, indicating that they increase the binding rate of RTA. Arginine residues form a positively charged patch to bind to negatively charged residues at the C-termini of P-proteins. When electrostatic interactions conferred by the arginines are lost, hydrophobic interactions are also abolished, suggesting that the hydrophobic interactions alone are insufficient to allow binding. We propose that Arg235 serves as an anchor residue and cooperates with nearby arginines and the hydrophobic interactions to provide the binding specificity and strength in ribosome targeting of RTA.

The plant toxin ricin produced by the castor bean plant, *Ricinus communis*, is one of the most potent and lethal substances known^1^. Due to its wide availability and the ease of production, ricin has been exploited as an agent of bioterrorism and biological warfare^1^,^2^. Tons of ricin are produced annually worldwide as a by-product of castor oil. Ricin induces apoptosis in transformed cells and has been used for chemotherapy in humans^3^,^4^. The related Shiga toxins (Stx) produced by *E. coli* (STEC) can cause severe morbidity and mortality, including hemorrhagic colitis (HC) and hemolytic uremic syndrome (HUS)^5^. These toxins remain a major challenge for food safety and public health. Currently, no FDA-approved vaccine or therapeutics exist to protect against ricin intoxication or Stx-mediated disease. Ricin is a type II ribosome inactivating protein (RIP) consisting of ricin toxin A chain (RTA) and ricin toxin B chain (RTB) connected by a disulfide bond^6^. RTB is a galactose specific lectin that binds to glycolipids or glycoproteins on the cell surface to promote endocytosis of the toxin^7^. RTA is an *N*-glycosidase that cleaves a conserved adenine (A4324 in rat and A3027 in yeast) from the α–sarcin/ricin loop (SRL) of the large rRNA^8^,^9^. Ricin holotoxin enters cells through RTB mediated endocytosis. In the ER, the disulfide bond between RTA and RTB is reduced, releasing RTA from RTB^10^. The free RTA enters the cytosol by the endoplasmic reticulum associated degradation (ERAD) pathway to reach the ribosome, to depurinate the SRL and inhibit protein synthesis^11^,^12^.

Though the SRL is highly conserved, the ribosomal context enhances the depurination rate by several orders of magnitude, indicating that ribosomal proteins determine the specificity of ricin^13^,^14^. We identified P-proteins of the ribosomal stalk as the host target of RTA^15^ and showed that RTA binds to P-proteins to depurinate ribosomes in human cells^16^. The deletion of P-proteins reduced depurination activity and cellular sensitivity to the toxin, indicating that binding to the P-protein stalk is a critical step in depurination of the SRL and in toxicity of ricin^15^. The ribosomal stalk and the SRL are part of the GTPase Associated Center (GAC) of the ribosome and stimulate the translation factor-dependent GTP hydrolysis^17^,^18^. The bL12/P-protein stalk represents the last structure on the ribosome for which the atomic architecture and function remain poorly established. In eukaryotes, the stalk is organized as a pentamer with ribosomal uL10 protein (former name P0)^19^, which anchors two eukaryotic unique P1/P2 heterodimers. The eukaryotic stalk proteins contain a highly conserved motif present at the C-terminal domain (CTD), consisting of a stretch of acidic and hydrophobic amino acids, involved in interaction with translational GTPases and RIPs^13^,^20^. The CTD of P1/P2 is unstructured and extends away from the dimerization domain^21^. RIPs such as trichosanthin (TCS), Shiga toxin 1 (Stx1) and maize RIP interact with the CTD of P-proteins to depurinate the SRL^22^,^23^,^24^,^25^,^26^.

The structure of a 11-mer peptide (P11) corresponding to the conserved last 11 residues of the stalk proteins [SDDDMGFGLFD] in a complex with TCS showed that the acidic amino acids at the amino end of P11 [DDD] form favorable electrostatic interactions with the positively charged Lys173, Arg174 and Lys177 of TCS, while the hydrophobic carboxyl end of P11 [FGLF] is docked into a hydrophobic pocket of TCS^25^. Acidic and hydrophobic regions of P11 were shown to represent two major anchors recognized by cationic and hydrophobic surfaces on the A1 chain of Stx1^23^,^24^. Recent structures of P11 and a 6-mer peptide (P6) corresponding to the conserved last 6 residues of the stalk proteins [GFGLFD] in a complex with RTA showed that the peptide docks into a hydrophobic pocket at the C-terminus of RTA^27^. The negatively charged [SDDDM] motif at the amino end of the P11 was not defined in the crystal. The structure revealed that Arg235 and Arg234 of RTA participate in the interaction. However, the importance of these arginines relative to other arginines at the RTA/RTB interface was not addressed^27^. The interaction of RTA with the P-protein peptide differed from that observed with TCS, indicating that RTA uses a novel binding mode to interact with the CTD of P-proteins^27^.

The different modes of interaction of RIPs with the stalk may represent a new target for the identification of RIP-specific inhibitors. However, the role of these interactions on the depurination activity and cytotoxicity of RTA in cells is not well understood. Molecular details of this interaction could provide new evidence for the mechanism of depurination by RIPs. We showed that association of RTA with the ribosome is a two-step process characterized by two kinds of electrostatic interactions. AB2 interactions, which are non-stalk dependent, salt sensitive and have slow association and dissociation rates and AB1 interactions, which are stalk-dependent, less salt sensitive and have faster association and dissociation rates^28^. AB1 interactions between RTA and yeast ribosomes are lost when P-proteins are deleted from the ribosome, suggesting that they represent the interaction of RTA with the P-proteins^28^.

Previous studies showed that catalytic activity of ricin decreases upon modification of arginine residues outside the active site cleft^29^ and upon site-directed mutagenesis of surface arginines^30^. Although the active site of RTA is exposed to the solvent in the holotoxin, the holotoxin does not inhibit protein translation because RTB covers the ribosome binding site on RTA. RTA is active only after it is released from RTB and after ribosome binding site is exposed^14^. There are seven arginine residues clustered at the RTA/RTB interface, indicating the existence of a positively charged surface (Fig. 1a,b). Arg234 is mostly covered by RTB. Arg189 and Arg235 are partially covered and Arg191, Arg193, Arg196 and Arg197 are exposed (Fig. 1a). We previously showed that R189A/R234A and R193A/R235A double mutations affected the electrostatic interactions of RTA with the ribosome^14^ and reduced the depurination activity and toxicity of RTA (ref. 14). Here to address the relative importance of each arginine at the RTA/RTB interface as an anchor for the CTD of ribosomal stalk proteins, we generated RTA constructs with single, double and triple mutations. These mutants were assessed for their ability to bind ribosomes, to depurinate the SRL and to cause toxicity in yeast. Our results indicate that mutations in interfacial arginines significantly reduce the catalytic activity and toxicity of RTA. Although the structural analysis showed that RTA interacts with P-proteins via hydrophobic rather than electrostatic interactions^27^, we show here that arginines at the RTA/RTB interface contribute to the fast electrostatic interactions with the ribosome. We present the first evidence that Arg235 serves as a key interacting residue and cooperates with nearby arginines to allow RTA to bind to the stalk with fast kinetics to achieve binding specificity and speed in depurinating the SRL.

## Results

### R235A has lower depurination activity and toxicity than the other single arginine mutants

**Figure 1:**
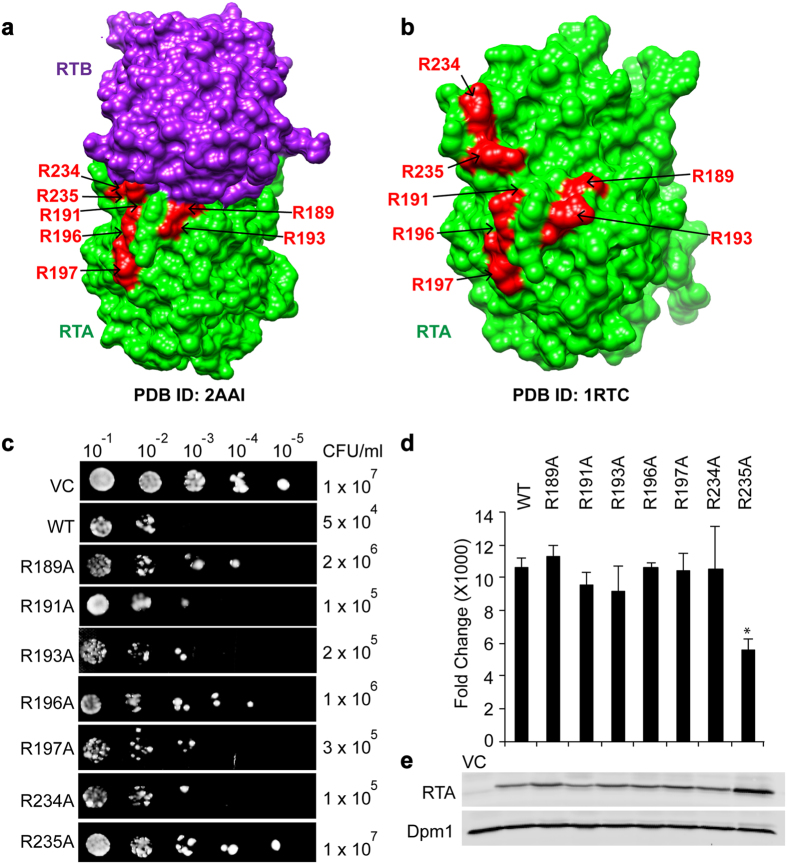
Toxicity and depurination activity of RTA mutants in yeast. (**a**) Structure of ricin holotoxin (PDB ID: 2AAI)^45^. RTA is colored green with arginine residues highlighted in red. RTB is colored purple. (**b**) Structure of RTA (PDB ID: 1RTC)^46^. RTA is colored green with arginine residues highlighted in red. (**c**) Viability of RTA mutants. Yeast cells were transformed with either WT RTA or RTA mutants and spotted on glucose at 4 hours post induction (hpi). (**d**) Ribosome depurination in yeast expressing RTA mutants at 3-hpi. Data was analyzed by one-way ANOVA to test for statistical differences between treatments and Bonferroni’s test was used to perform pairwise comparisons (*P < 0.05). (**e**) RTA expression in yeast at 4 hpi. Total protein from yeast cells (1 OD) was loaded on a 15% SDS-PAGE gel, transferred to nitrocellulose and probed with monoclonal antibody against RTA. Anti-dolichol phosphate mannose synthase (Dpm1) was used as loading control. Cells transformed with the empty vector were used as vector control (VC). The blots were imaged using Odyssey CLx Infrared Imaging System (LI-COR Biosciences, Lincoln, NE). Cropped image of the blot is shown. Full length image is included in Fig. S4.

To identify the contribution of each arginine at the RTA/RTB interface to the toxicity of the toxin, Arg189, Arg191, Arg193, Arg196, Arg197, Arg234 and Arg235 were mutated to alanine and their cytotoxicity was analyzed in yeast in comparison to wild type (WT) RTA. The mutant genes were cloned into a low copy plasmid (pRS415) downstream of a *GAL1* promoter. Viability assay showed reduced toxicity of all mutants compared to WT at 4 hours post induction (hpi). Viability of R235A was similar to the vector control (VC) (Fig. 1c). Yeast expressing WT RTA showed longer doubling time (Fig. S1) compared with yeast harboring the vector (VC) on glucose, indicating that cell growth was affected due to leaky expression of the toxin. Doubling times of R189A, R191A, R193A, R196A, R197A and R234A were between WT and VC, indicating these mutants had reduced toxicity compared to WT RTA, but were more toxic than VC (Fig. S1). The doubling time of R235A was similar to VC, indicating reduced toxicity compared to the other single mutants (Fig. S1). Analysis of depurination using qRT-PCR^31^ indicated that only R235A showed significant reduction in depurination at 3 hpi (Fig. 1d). In contrast, differences in the level of depurination between the other single mutants and WT were not significant. Immunoblot analysis showed expression of all mutants in yeast (Fig. 1e). The least toxic mutant, R235A was expressed at the highest level (Fig. 1e), indicating that the level of expression correlated inversely with toxicity^14^. These results showed that among the single arginine mutants, R235A has the lowest toxicity and depurination activity.

### Arginine mutants depurinate the SRL similar to WT RTA, but depurinate ribosomes less than WT RTA

**Figure 2:**
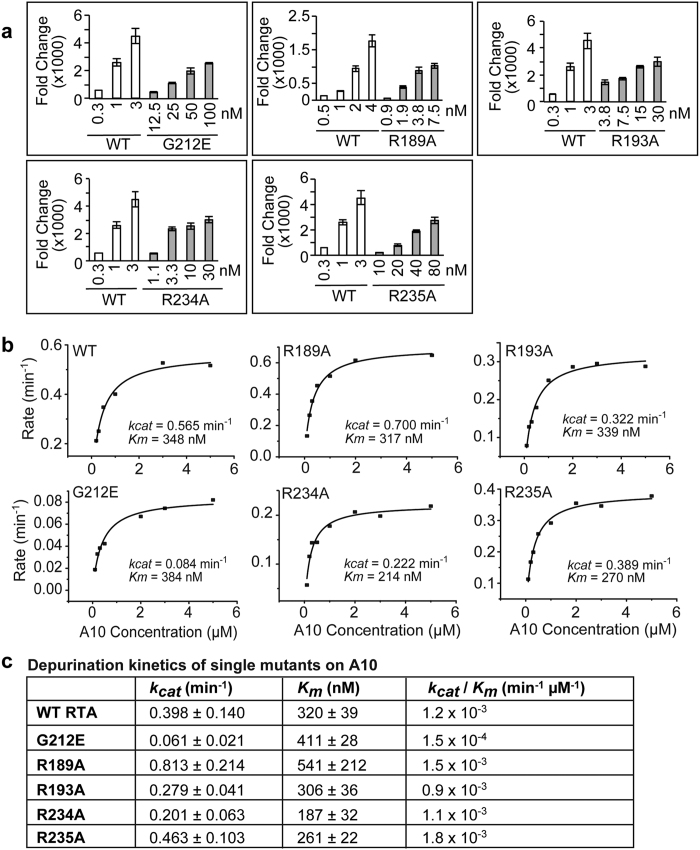
Depurination of ribosomes and RNA by single arginine variants. (**a**) Depurination of yeast ribosomes by WT RTA and RTA variants. Yeast ribosomes (4 pmoles) were mixed with indicated concentrations of WT or mutant RTA and incubated at 19 °C for 5 min. Depurination level was measured by qRT-PCR. (**b**) Michaelis-Menten fits of depurination of A10 by WT RTA and RTA mutants. A10 (0.1–5 μM) was incubated with 30 nM WT RTA or RTA mutants. G212E was used as a control. Each plot was from a representative experiment. Results from three independent experiments were summarized in (**c**). (**c**) The *k*_cat_ and *K*_m_ were calculated by fitting the plot with Michaelis-Menten model using Origin 8.6 (OriginLab Corp. Northampton, MA). The analysis was repeated three times. Error bars represent S. E. where n = 3 independent experiments.

To determine the relative contribution of individual arginines towards the depurination activity of RTA, we expressed R189A, R193A, R234A and R235A in *E. coli* and purified them. We examined their depurination activity on purified yeast ribosomes *in vitro* (Fig. 2a). G212E, which carried a mutation near the active site, was used as a control. G212E, R189A, R193A, R234A and R235A depurinated yeast ribosomes at a 100-, 4-, 15-, 10- and 80-fold lower level than WT, respectively (Fig. 2a), indicating that R235A showed the lowest activity compared with the other arginine mutants.

To determine if arginine mutations affected the catalytic activity of the mutants, we examined depurination kinetics of the mutants on a 10mer RNA stem loop mimic of the SRL (A10) using a luminescent assay^14^,^32^ (Fig. 2b). Kinetic parameters from three independent experiments are summarized in Fig. 2c. WT RTA had a *k*_cat_ of 0.398 min^−1^ and G212E had a *k*_cat_, which was 6-fold lower than WT RTA on A10. R193A, R234A and R235A had similar *k*_cat_ as WT while *k*_cat_ of R189A was 2-fold higher than WT. The catalytic efficiencies (*k*_*cat*_*/K*_*m*_) of arginine mutants were similar to WT RTA, but *k*_*cat*_*/K*_*m*_ of G212E was 10-fold lower due to a mutation near the active site. These data show that arginine mutants have similar catalytic efficiency as WT RTA, indicating that they do not affect the catalytic activity or the electrostatic interactions of RTA with the SRL. Therefore, the reduction in their depurination activity on intact ribosomes is not due to reduced catalytic activity.

### Arginine mutations disrupt the interaction between RTA and the ribosome

**Figure 3:**
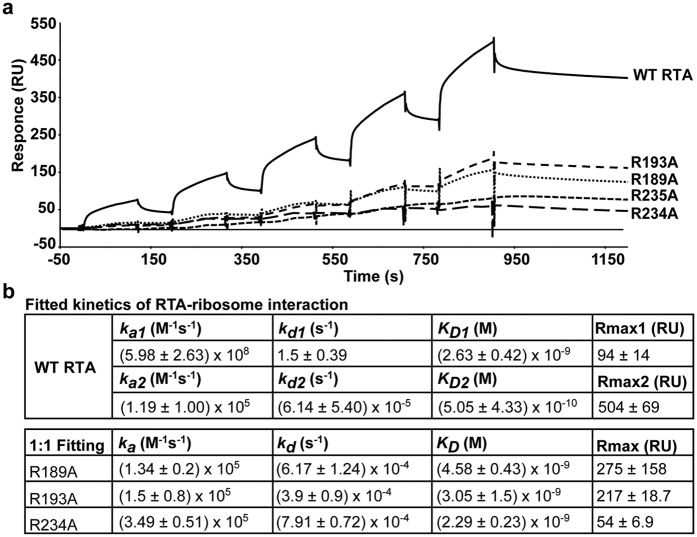
Interaction between WT and mutant RTA and yeast ribosomes. (**a**) RTA mutants, R189A, R193A, R234A and R235A were captured on an NTA chip at 450 RU. Purified yeast ribosomes were passed over the surface of the chip as analyte at five different concentrations (5, 10, 20, 40, 80 nM). (**b**) Kinetic constants calculated from three independent experiments.

To determine if arginine mutations affected ribosome binding, we examined the interaction between the single arginine mutants and yeast ribosomes using surface plasmon resonance (SPR) with Biacore T200 (Fig. 3a). WT RTA and single arginine mutants were captured on an NTA chip at 450 RU. Purified yeast ribosomes were passed over the surface of the chip as the analyte. For each mutant, ribosomes were injected at 5 different concentrations (5, 10, 20, 40, 80 nM) using single kinetic injection method with a short dissociation time between each injection. The single mutants bound yeast ribosomes at a lower level than WT RTA (Fig. 3a). The interaction of WT RTA and the ribosome did not fit a simple 1:1 interaction model, but fit to the heterogeneous model. Fitting analysis showed that WT RTA bound to the ribosome with two kinds of interactions: one (AB1) with fast association rate (*k*_a1_) at 5.98 × 10^8^ M^−1^s^−1^ and fast dissociation rate (*k*_d1_) at 1.5 s^−1^, and another (AB2) with slow association rate (*k*_a2_) at 1.19 × 10^5^ M^−1^s^−1^ and slow dissociation rate (*k*_d2_) at 6.14 × 10^−5^ s^−1^ (Fig. 3b). In contrast, the interaction of R189A, R193A and R234A with the ribosome fit 1:1 binding model and showed only slow association and slow dissociation similar to the AB2 interactions of WT RTA (Fig. 3b). R235A bound the ribosome at the lowest level and showed almost no dissociation. Thus we could not fit the signal from R235A to any model. These results demonstrated that arginine mutations reduced the interaction of RTA with the ribosome primarily through the loss of AB1 interactions. R235A mutation showed the greatest reduction, indicating that Arg235 makes the largest contribution to the AB1 interactions with the ribosome. R189A, R193A and R234A all showed a reduction compared to WT RTA, though to a lesser extent than R235A, indicating Arg189, Arg193 and Arg234 also contribute to the AB1 interactions.

### Nearby arginines cooperate with R235A to further reduce the depurination activity and toxicity of RTA

**Figure 4:**
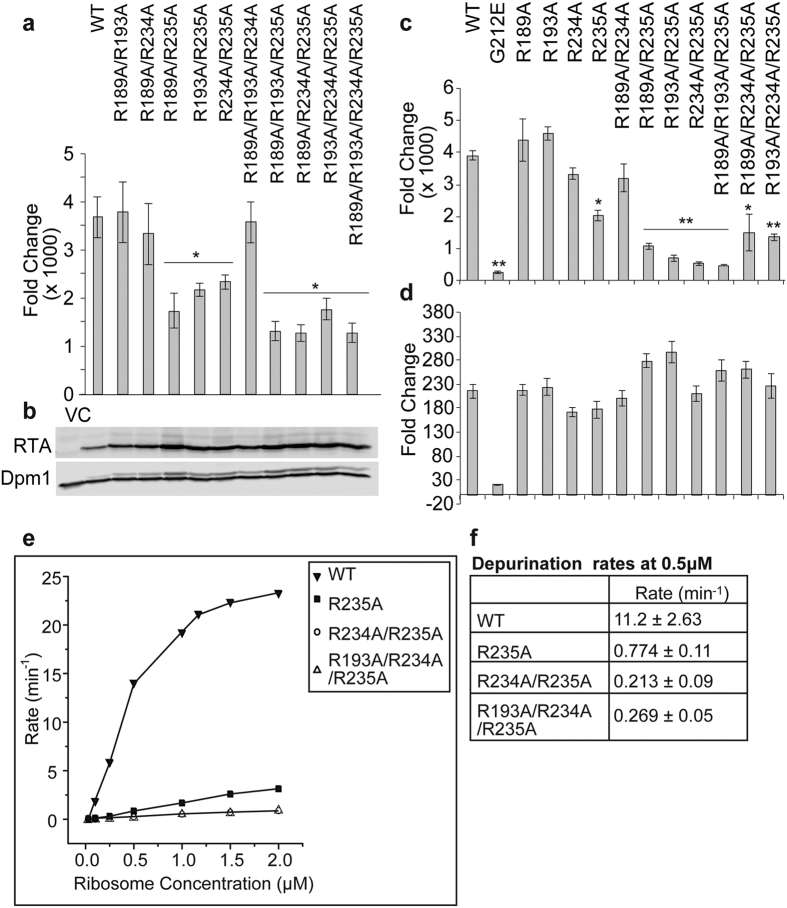
Depurination of ribosomes and RNA by double and triple arginines mutants. (**a**) Ribosome depurination in yeast by double and triple mutants at 3 hpi quantified by qRT-PCR. Data were analyzed by one-way ANOVA to test for statistical differences between treatments and Bonferroni’s test was used to perform pairwise comparisons (*P < 0.05). (**b**) Protein expression level in yeast at 4 hpi. Total protein from yeast cells (1 OD) was separated on 15% SDS-PAGE and probed with monoclonal anti-RTA. Monoclonal anti-Dpm1was used as loading control. The blots were imaged using Odyssey CLx Infrared Imaging System (LI-COR Biosciences, Lincoln, NE). Cropped image of the blot is shown. Full length image is included in Fig. S5. (**c**) Depurination of yeast ribosomes by double and triple mutants. Yeast ribosomes (4 pmoles) were mixed with each double or triple mutant (20 nM) or WT RTA (4 nM) and incubated at 30 °C for 5 min. Depurination was quantified using qRT-PCR. Data were analyzed using PROC MIXED to test for statistical differences between treatments and multiple comparisons between treatment means were made using an approximate t-test, corrected using the Bonferroni adjustment option. (*P < 0.05, **P < 0.01). (**d**) Depurination of total RNA from yeast. Total RNA (1 μg) was mixed with WT or mutant RTA (300 nM) and incubated at 30 °C for 30 min. Depurination level was measured by qRT-PCR. (**e**) Rate of depurination of yeast ribosomes (0.1–2 μM) by WT RTA, R235A, R234A/R235A and R193A/R234A/R235A. Each plot is from a representative experiment. (**f**) Average rate of depurination at 0.5 nM ribosome concentration was calculated from three independent experiments. Error bars represent S. E. where n = 3 replicates.

To determine if combining two or more arginine mutations can disrupt the ribosome interaction and further reduce the depurination activity and toxicity of RTA, we made different combinations of double, triple and quadruple arginine mutations and expressed them in yeast. Growth curves in glucose medium showed reduced toxicity for all multiple arginine mutants and those containing R235A had similar growth curve and doubling time as VC (Fig. S1). Viability assays showed that among the triple arginine mutants those containing R235A reduced toxicity more than those without R235A (Fig. S2). Analysis of depurination (Fig. 4a) showed that R235A containing double, triple and quadruple mutants showed significantly reduced depurination level compared to WT at 3 hpi. In contrast, the double and triple mutants that did not contain R235A showed similar level of depurination as WT RTA at 3 hpi. We did not observe significant differences in depurination among the double or triple mutants containing 235A. Immunoblot analysis showed that all mutants were expressed in yeast (Fig. 4b).

To determine how multiple arginine mutations affected the catalytic activity of RTA, we purified four double mutants (R189A/R234A, R189A/R235A, R193A/R235A and R234A/R235A) and three triple mutants (R189A/R193A/R235A, R189A/R234A/R235A and R193A/R234A/R235A) and examined their depurination activity on yeast ribosomes (Fig. 4c). The results showed that R235A had significantly lower toxicity and depurination activity compared with the other single arginine mutants. The double or triple mutants containing R235A were significantly less active than those that did not contain R235A (Fig. 4c).

To determine if reduced toxicity is due to altered catalytic activity on RNA, we tested depurination activity of the mutants on total yeast rRNA. The G212E variant showed the lowest activity on naked RNA (Fig. 4d). In contrast, single, double and triple arginine mutants depurinated yeast rRNA at a similar level as WT RTA. These data showed that although the depurination activity of multiple arginine mutants was reduced on yeast ribosomes, arginine mutations did not affect their catalytic activity on RNA. We did not observe detectable binding when we examined the interaction of double and triple mutants with yeast ribosomes using SPR.

To understand the differences in ribosome depurination by single, double and triple mutants, we examined the depurination kinetics of WT, R235A, R234A/R235A and R193A/R234A/R235A on yeast ribosomes using a luminescent assay (Fig. 4e)^14^,^32^. WT RTA showed increased depurination rate as ribosome concentration increased and started to saturate at high ribosome concentrations. Arginine mutants also showed increased reaction rate at higher ribosome concentrations but the curves were linear and did not reach saturation, indicating increased *K*_*m*_. To compare activities of the mutant proteins, we calculated the rate of depurination of each mutant at 0.5 μM ribosome concentration from three independent experiments (Fig. 4f). The depurination rates of R235A, R234A/R235A and R193A/R234A/R235A were more than 14-, 52- and 41-fold lower than WT RTA, respectively. The depurination rates of the double and triple mutants were similar and 3-fold lower than R235A. These data showed that Arg235 is critical for RTA to bind the ribosome and to depurinate the SRL. Combination of Arg235 with one more arginine at the RTA/RTB interface contributes to complex formation by further enhancing the depurination rate of RTA.

We used VASP-E (Volumetric Analysis of Surface Properties with Electrostatics)^33^ to infer the contribution of each RTA residue to electrostatic complementarity in the RTA-P6 complex^27^. VASP-E, explained further in the methods section, infers residue-level contributions by estimating changes in complementarity that occur when the electrostatic potential of an amino acid is removed. If electrostatic complementarity increases when the potential of an amino acid is removed, then the charge of that amino acid normally reduces complementarity. Conversely, if complementarity decreases when the potential of an amino acid is removed, that residue normally increases complementarity. While changes in electrostatic complementarity do not determine affinity, they support a testable hypothesis for the role of these amino acids.

**Figure 5:**
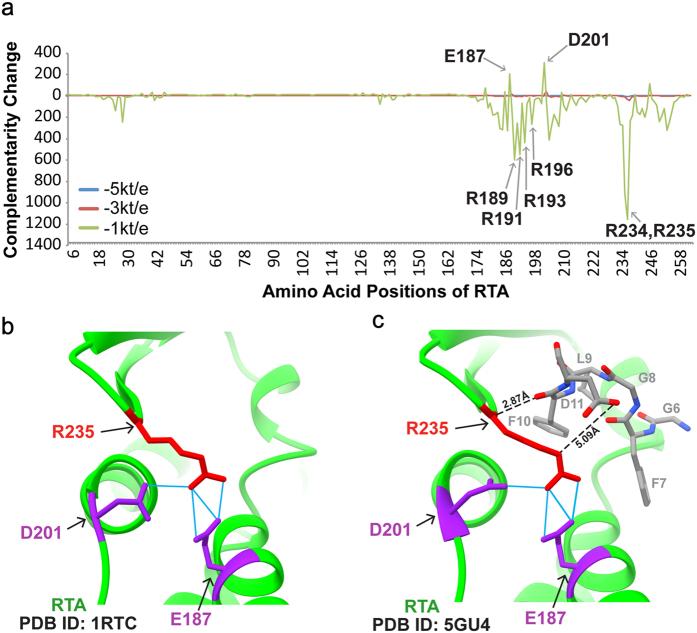
Structure alignment and VASP-E analysis. (**a**) Changes in electrostatic complementarity in the RTA-P6 complex (PDB ID: 5GU4) caused by individually removing the electrostatic contribution of each amino acid in RTA. Blue, red, and green lines plot changes in complementarity measured at different isopotential thresholds. Bonds formed between Arg235 and nearby residues, Glu187 and Asp201 in RTA (PDB ID: 1RTC)^46^ and RTA-P6 complex (PDB ID: 5GU4)^27^ are shown in (**b**) and (**c**) respectively. RTA is shown as green ribbon. Arg235 is colored in red. Glu187 and Asp201 are colored in purple. P6-peptide is shown as grey sticks with nitrogen and oxygen atoms highlighted in blue and red, respectively. Salt bridges between Arg235 and Glu187 and a hydrogen bond Between Arg235 and Asp201 are represented as solid blue lines. Hydrogen bonds between Arg235 and Asp11 are represented as dashed black lines.

The change in electrostatic complementarity caused by the removal of the electrostatic potential of individual RTA residues is plotted in Fig. 5a. Adjacent downward spikes at Arg234 and Arg235 suggest that the omission of R234 and R235 radically reduces electrostatic complementarity, supporting the idea that alanine substitutions reduce complementarity as well. Upward spikes at Glu187 and Asp201 suggest that their omission will increase complementarity. As shown in Fig. 5b and c, Arg235 can form a salt bridge with Glu187 and a hydrogen bond with Asp201 in both free RTA (PDB ID: 1RTC) and RTA-P6 complex (PDB ID: 5GU4). These interactions may reduce the electrostatic interaction of RTA with P6. Between Glu187 and Asp201, arginines 189, 191, 193 and 196 also contribute to electrostatic complementarity. Arg197 was not picked possibly because the N-terminal [SDDDM] motif was not defined in the structure^27^. Together, these data underpin the rationale for our experiment because they suggest that the arginines at the interface, especially Arg234 and Arg235, contribute to an attractive electrostatic interaction with P6, and that losing this attraction by mutation could reduce P6-RTA affinity.

## Discussion

Previous studies showed that electrostatic interactions are critical for binding of RTA to intact ribosomes and targeting of a ribotoxin to the SRL^28^,^34^. Arginine residues, which create a positively charged surface on Stx1 and TCS, were shown to be important for the interaction with the anionic residues at the CTD of P-proteins^24^,^25^. Similarly, lysine residues in the maize RIP were critical for the interaction with the anionic residues at the CTD of P2 protein^35^. Although RTA and TCS have similar structures and interact with the CTD of P-proteins, the crystal structure of RTA-P6 complex (PDB ID: 5GU4) adopts a different orientation than the TCS-P11 complex^27^. In the crystal structure P6 peptide [GFGLFD] docks into a pocket formed by six hydrophobic residues (Tyr183, Leu207, Phe240, Ile247, Pro250 and Ile251) and five polar residues (Gln182, Ser203, Gln233, Arg234 and Arg235) in RTA^27^. The RTA-P6 interaction was mainly hydrophobic with Phe10 and Asp11 of C6-P2 forming hydrogen bonds with Arg234 and Arg235 of RTA, respectively. Although the amino end of the peptide [SDDDM] was not defined in the crystal, it was found to increase the binding affinity of P11 and was proposed to bind to Arg189 and Arg193 of RTA^27^. In contrast, the crystal structure of P11 [SDDDMGFGLFD] with TCS (PDB ID: 2JDL)^25^ showed that Asp4 forms salt-bridges with Lys173 and Arg174 of TCS and Asp2 forms a hydrogen bond with Gln169 of TCS^25^. The importance of the DDD motif in the interaction of TCS with P2 proteins was shown by mutagenesis^22^. Removal of the acidic amino acids [SDDD] from P11 resulted in a peptide [MGFGLFD], which did not interact with RTA or Stx1^23^.

We previously reported that double mutations at arginine residues at the RTA/RTB interface led to a significant increase in the *K*_m_ and a decrease in *k*_cat_ toward the ribosome without affecting the *K*_m_ or *k*_cat_ towards an SRL mimic RNA, indicating that electrostatic contacts contribute to the interaction of RTA with the ribosome^14^. Analysis of binding kinetics showed that the electrostatic interactions of RTA with the ribosome follow a two-step binding model^28^. We proposed that the slower non-stalk dependent electrostatic interactions (AB2) concentrate RTA molecules on the ribosome to reduce the search space and to allow the faster electrostatic interactions with the stalk P-proteins (AB1)^28^. Here to test this model and to identify the relative contribution of each arginine, we mutated every arginine at the RTA/RTB interface. WT RTA bound ribosomes with fast association (5.98 × 10^8^ M^−1^s^−1)^ and dissociation rates (1.5 s^−1^), which were close to the limit of detection of Biacore T200 (10^3^ to 3 × 10^9^ M^−1^s^−1^ for association and 10^−5^ to 1 s^−1^ for dissociation). The actual rates might be even higher and reach the limit of protein-protein association rates (10^9^ M^−1^s^−1^)^36^. We show here that single mutations at Arg189, Arg193, Arg234 and Arg235 at the RTA/RTB interface eliminated AB1 interactions of RTA with the ribosome (Fig. 3), demonstrating that these arginines are critical for maintaining the fast association and dissociation rates of the interaction with the stalk P-proteins. The positively charged arginines increased the rate of association of RTA with the ribosome over 1000-fold and rate of dissociation over 10,000-fold (AB1), indicating that arginines affect the specificity of the interaction between RTA and the stalk by enhancing protein recognition. Since the eukaryotic stalk consists of one copy of the P0 protein and two copies of the P1/P2 dimers with conserved CTD, the pentameric organization of the stalk may contribute to the high association rate possibly by binding to more than one RTA^28^. These results provide support for our model and suggest that the electrostatic interactions with P-proteins allow RTA to achieve binding specificity and speed in depurinating the SRL.

In the RTA-P6 crystal structure, Arg234 and Arg235 participate in the interaction with Phe10 and Asp11 of P11, respectively^27^. We show here Arg235 has a different and more important role compared with the other arginine residues. R235A mutant was the least toxic among the single mutants in yeast (Fig. 1c) and the least active in depurinating yeast ribosomes (Fig. 1d). The R235A mutation did not affect the active site of RTA because it depurinated the SRL mimic RNA with a similar *k*_cat_/*K*_m_ as WT RTA (Fig. 2c). Alanine substitution, which eliminated the positive charge of Arg235, led to the greatest reduction in the faster electrostatic interactions with the ribosome (AB1) (Fig. 3a). The predictions of VASP-E analysis were supported by our experimental results indicating that Arg235 contributes to an attractive electrostatic interaction with P11 (Fig. 5a). These data indicate that Arg235 is the most critical arginine at the RTA/RTB interface for RTA-stalk interaction, depurination activity and toxicity.

Point mutations at Arg189, Arg191, Arg193, Arg196, Arg197, Arg234 and Arg235 reduced the toxicity of RTA (Fig. S1). The double and triple mutants that contained the R235A mutation showed significant reduction in toxicity, depurination activity and ribosome binding compared to the single R235A mutant (Fig. S2, Fig. 4a,c), indicating that one anchor is not enough, arginines near Arg235 also contribute to the RTA/stalk interaction. Since double and triple arginine mutants depurinated naked RNA similar to WT (Fig. 4d), the reduction in toxicity and depurination activity on ribosomes was not likely due to a reduction in the catalytic activity. In the crystal structure of RTA (PDB ID: 1RTC) Arg234 and Arg235 are located away from Arg189, Arg191, Arg193, Arg196 and Arg197. The α-carbon of Arg193 and Arg235 are 19 Å apart. Since double mutations at R193A/R235A and R189A/R234A did not affect the structure of RTA^14^, it is unlikely that the addition of a third mutation will disrupt the ribosome binding site. Consistently, we showed that triple mutants were not significantly different from double mutants in ribosome depurination activity (Fig. 4a,c). We were not able to detect binding of double and triple mutants to the ribosome, suggesting that the reduction of toxicity and depurination activity was due to weakened ribosome binding. These data demonstrate that Arg235 is the key interacting residue and nearby arginines facilitate the interaction of Arg235 with the ribosome.

Sequence alignment showed that the LF motif at CTD of stalk proteins is conserved among eukaryotes, including yeast, mice, humans and plants, such as *Ricinus communis* where ricin is naturally produced (Fig. S3). The hydrophobic residues at the C-terminus of P11, especially the conserved LF motif docks into a hydrophobic pocket of RTA^27^, TCS^25^ and the elongation factors^37^, indicating hydrophobic interactions are also a major binding force. However, when P7 was fused with calmodulin it could not be pulled down by RTA^23^. In contrast, when P6 or P7 were fused to GST, they were pulled down by RTA^27^. These conflicting results may be because hydrophobic interactions alone are insufficient to bring RTA and the peptide together. Additional forces may be necessary for the interaction between the LF motif and RTA. A point mutation at Arg235 or at a nearby arginine eliminated the fast interactions of RTA with the ribosome. When multiple arginines were mutated, we could not detect any interaction, suggesting that without a positive charge on arginines the hydrophobic interactions are abolished. Although the structure demonstrated that hydrophobic interactions play a major role in RTA/stalk interaction^27^, our data suggest that electrostatic interactions supersede formation of the hydrophobic interactions. Arg235 cooperates with other arginine residues at the RTA/RTB interface to provide long distance electrostatic forces to overcome diffusion and geometric constraints of the binding site, allowing stronger hydrophobic interactions to take place. Arg189, Arg191, Arg196 and Arg197 likely interact with Asp2-4 of the P11 peptide. These interactions may not be observed in the structure due to their highly dynamic and transient nature, since the structure represents a static view of a series of transient interactions^27^.

VASP-E analysis predicted that although Arg234 and Arg235 made the largest contribution to the electrostatic interactions of RTA with P6, Arg189, Arg191, Arg193, Arg196 and possibly Arg197 also increased the electrostatic complementarity (Fig. 5a). Glu187 and Asp201 reduced the electrostatic complementarity with P6, suggesting that Arg235 is constrained by Glu187 and Asp201 in RTA. As shown in Fig. 5b,c Arg235 forms salt bridges with Glu187 and a hydrogen bond with Asp201 in both free RTA and in the RTA-P6 complex^27^, suggesting that Glu187 and Asp201 may keep Arg235 in the optimal conformation for forming a hydrogen bond with Asp11. The constraint on Arg235 may provide the critical stability required for specific recognition between RTA and the ribosomal stalk. Based on the RTA-P6 structure, the strong effect of the R235A mutation is due to the loss of electrostatic interactions with Asp11^27^. We show here that electrostatic interactions make a strong contribution to the interactions of RTA with the ribosome and work together with the hydrophobic interactions to provide the critical binding specificity and strength in ribosome targeting of RTA. Identification of Arg235 as an anchoring residue provides new insights into the mechanism of ribosome inactivation and valuable information for the drug design process. The results reported here are likely to serve as a starting point in the generation of RIP antidotes.

## Methods

### Protein structure visualization

All protein structures were downloaded from the Protein Data Bank (PDB)^38^ and visualized using the UCSF Chimera package (https://www.cgl.ucsf.edu/chimera/download.htmL)^39^.

### Plasmid and cloning

Site-directed mutagenesis was used to introduce mutations into RTA in pBluescript (NT855). The mutations were confirmed by sequencing. The wild type and mutated mature RTA genes were cloned into yeast expression vector pRS415 with *LEU2* selective marker under the control of *GAL1* promoter to generate WT (NT1622), R189A (NT1623), R191A (NT1624), R193A (NT1625), R196A (NT1626), R197A (NT1627), R234A (NT1628), R235A (NT1629), R189A/R193A (NT1630), R234A/R235A (NT1631), R189A/R235A (NT1632), R191A/R235A (NT1633), R196A/R235A (NT1634), R189A/R234A (NT1635), R191A/R196A (NT1636) and R193A/R235A (NT1637), R189A/R193A/R234A (NT1638), R189A/R234A/R235A (NT1639), R193A/R234A/R235A (NT1640), R189A/R193A/R235A (NT1641) and R189A/R193A/R234A/R235A (NT1642). The empty vector (NT1616) and WT and mutant RTA constructs were transformed into *S. cerevisiae* strain W303 (*MATa ade2-1 trp1-1 ura3-1 leu2-3, 112 his-3-11, 15 can1-100*).

### Viability assay

Yeast (W303) cells containing RTA constructs were grown at 30 °C in synthetic dropout (SD-LEU) medium supplemented with 2% glucose overnight and expression was induced by transferring them to SD-LEU medium with 2% galactose. Cells were collected at 4 and 8 hours post induction and serial dilutions of 0.1 OD_600_ were plated on SD-LEU plates containing 2% glucose. Plates were incubated for 2–3 days at 30 °C.

### Growth curve analysis

Yeast (W303) cells transformed with WT RTA or RTA mutants were grown in SD-LEU medium containing glucose overnight. The overnight culture was diluted in SD-LEU medium containing glucose and grown for 36 hours at 30 °C in an Eon plate reader (BioTek Instruments, Inc. Winooski, VT, USA). Doubling time was calculated from the exponential phase of each curve.

### Immunoblot analysis

The expression of toxins was induced for 4 hours before sampling. Total protein was extracted as described^40^. Yeast cells (OD_600_ of 3) were washed with 2 M LiAc for 5 min on ice then spun down at 6000 g. The pellet was resuspended in 0.5 mL of 0.4 M NaOH for 5 min on ice and then spun down again at 6000 g. Next, they were washed with 0.5 mL of 100 mM Tris-HCl pH 6.8 and spun down again. The pellet was resuspended in 2X SDS (10 μL/OD), heated at 95 °C for 5 min and incubated for 1 h at 30 °C. The extract was spun down again and 10 μL of the supernatant was loaded on a 15% gel. The separated protein was transferred to nitrocellulose membrane and probed with PB10^41^,^42^, monoclonal antibody against ricin. Anti-dolichol phosphate mannose synthase (Dpm1) (Life Technologies, Grand Island, NY, USA) was used as loading control^14^.

### RTA protein expression and purification

The genes encoding mature WT RTA and RTA mutants were cloned into the *E. coli* expression vector, pET28, which contains 10X His tag at the N-terminus to generate WT (NT1430), R189A (NT1679), R193A (NT1586), R234A (NT1680), R235A (NT1587), R189A/R193A (NT1681), R234A/R235A (NT1682), R189A/R235A (NT1683), R189A/R234A (NT1484), R193A/R235A (NT1414), R189A/R234A/R235A (NT1685), R193A/R234A/R235A (NT1686) and R189A/R193A/R235A (NT1588). WT RTA was purified using Ni-NTA agarose from QIAGEN (Valencia, CA, USA) as described^28^ and RTA mutants were purified by Dr. Karen Chave at the Northeast Biodefense Center protein expression core facility.

### Interaction of RTA proteins with yeast ribosomes

WT RTA and RTA mutants were captured on a Biacore T200 NTA chip through an N-terminal His-tag at 450 RU and the same amount of R193A/R235A mutant, which does not bind the ribosome^14^, was captured on the reference channel. Purified yeast ribosomes were passed over the surface of the chip at 40 μL/min as analyte at 5 different concentrations (5, 10, 20, 40 and 80 nM) for 2 min followed by 5 min dissociation using the single injection kinetic method^28^. The chip was regenerated after each round of injection and dissociation. Running buffer contained 10 mM HEPES, 150 mM NaCl, 50 μM EDTA, 0.003% P20 and 5 mM MgCl_2_. The regeneration solution contained 350 mM EDTA pH 8.3.

### Ribosome depurination

Ribosome depurination was quantified using qRT-PCR as previously described^31^. Briefly, for *in vivo* depurination, RNA was extracted from yeast cells using an RNase Mini Kit from QIAGEN (Valencia, CA, USA). For *in vitro* depurination, yeast ribosomes (4 pmoles) were mixed with indicated WT or mutant RTA in reaction buffer (60 mM KCl, 10 mM Tris-HCl, and 10 mM MgCl_2,_ pH7.4) and incubated at 19 °C for 5 min. RNA was purified using phenol/chloroform extraction followed by ethanol precipitation. RNA (375 ng) from each sample was used for reverse transcription using a High Capacity cDNA Reverse Transcription Kit from Applied Biosystems (Thermo Fisher, Waltham, MA). The 1:100 diluted reverse transcription products (4 μL) were used for real-time PCR. Depurination primers (5′-CTATCGATCCTTAGTCCCTCG-3′, 5′-CCGAATGAACTGTTCCACA-3′) were used to amplify depurinated rRNA. 25S rRNA was used as endogenous control.

### Ribosome and SRL depurination kinetics

A method described by Sturm and Schramm^32^ and modified by Li *et al*.^14^, was used to measure depurination kinetics. A 10mer RNA stem loop mimic of the SRL (5′-rCrGrCrGrArGrArGrCrG-3′) (A10) was obtained from Integrated DNA Technologies, Inc. (Coralville, IA, USA). RTA was used at 30 nM to depurinate 5 μM, 3 μM, 2 μM, 1 μM, 0.5 μM, 0.3 μM, 0.2 μM and 0.1 μM A10. The reaction was started by mixing the toxin and RNA at 37 °C in reaction buffer (10 mM Citrate, PH 4.0) at final volume of 70 μl followed by sampling 10 μl from each reaction every 1 min. Samples were mixed with 10 mL 2x coupling buffer on ice to quench the depurination reaction, 20 μL of ATPlite 1step luminescence reagent (PerkinElmer, Waltham,MA) was added. Luminescence signal was measured by Synergy 4 plate reader (BioTek Instruments, Inc. Winooski, VT, USA).

For ribosome depurination the continuous assay was used. Ribosomes purified from W303^15^,^28^ were desalted using a Zeba^TM^ Spin Desalting Column 40K MWCO (Thermo Fisher Scientific Inc, Rockford, IL, USA). Ribosomes were used at 0.1–2 μM and WT RTA, R235A, R234A/R235A, R193A/R234A/R235A were used at 1, 10, 20 and 50 nM, respectively. The reaction was started by adding toxin to the reaction mixture in 50 μL final volume. The background signal from ribosomes was subtracted. Adenine standard was measured in each experiment under the same conditions. Rates were calculated as described^14^.

### VASP-E analysis

VASP-E^33^ infers the contribution of RTA residues by estimating changes in electrostatic complementarity. First, DelPhi^43^ is used to individually evaluate the electrostatic potential fields of RTA and P6. Second, for a given isopotential threshold **n**, VASP-E is used to generate isopotentials at +**n** kT/e and −**n** kT/e on both RTA and P6. These isopotentials surround closed regions with opposite charges nearby each protein. Finally, VASP-E measures V1, the intersecting volume between the +**n** kT/e isopotential of RTA and the −**n** kT/e isopotential of P11 and V2, the intersecting volume between the −**n** kT/e isopotential of RTA and the +**n** kT/e isopotential of P6. Together, we regard the C, the sum V1 + V2, as a proxy for electrostatic complementarity between RTA and P6: Larger values of C suggest greater complementarity and smaller values suggest the opposite. This computation was performed at three different values for **n**, equal to 1 kt/e, 3 kt/e, and 5 kt/e, in order to sample the electrostatic field at a wide range of potentials. Regions with stronger positive or negative charge are surrounded by regions of weaker charge, so they necessarily have smaller volumes of intersection. Therefore, since computations that use **n** equal to 3 kt/e and 5 kt/e did not reveal notable variations in electrostatic complementarity (Fig. 5a), further calculations with larger **n** were not performed.

To evaluate the contribution of some RTA residue **a**, the process above is repeated without the contribution of **a**. This omission changes RTA isopotentials in the process above and leads to a different complementarity score C(a). The difference, D = C-C(a), can be used to infer the contribution of **a** to electrostatic complementarity: if D is negative, then omitting a increases complementarity, so the normal presence of **a** reduces it. If D is positive, then the opposite occurs.

### Statistical analysis

Data for Figs 1d and 4a were analyzed using one-way ANOVA (Analysis of variance) in Origin v. 8.6 (OriginLab Corp. Northampton, MA). Data in Fig. 4c were analyzed by mixed model analysis using PROC MIXED in SAS 9.4 (SAS Institute, Inc., Cary, NC) to test for statistical differences between treatments. Treatment effects were considered fixed and blocks (separate qRT-PCR plates) were considered random effects^44^. Least square means were calculated and multiple comparisons between treatment means were tested using an approximate t-test, corrected using the Bonferroni adjustment option.

## Additional Information

**How to cite this article**: Zhou, Y. *et al*. Ricin uses arginine 235 as an anchor residue to bind to P-proteins of the ribosomal stalk. *Sci. Rep.* **7**, 42912; doi: 10.1038/srep42912 (2017).

**Publisher's note:** Springer Nature remains neutral with regard to jurisdictional claims in published maps and institutional affiliations.

## Supplementary Material

Supplementary Information
