# Supplementary material for: Ricin uses arginine 235 as an anchor residue to bind to P-proteins of the ribosomal stalk
Source: Sci Rep. 2017 Feb 23;7:42912. doi: 10.1038/srep42912 (PMC5322317; doi:10.1038/srep42912)
Supplement: Supplementary Information [file srep42912-s1.pdf]

Supplementary Information

Ricin uses arginine 235 as an anchor residue to bind to P-proteins of the ribosomal stalk

Yijun Zhou, Xiao-Ping Li, Brian Chen and Nilgun E. Tumer

Group 1	Average	SD	Fold Change
VC	4.34	0.09	1.00
WT	6.20	0.15	1.43
R189A	5.45	0.04	1.26
R193A	5.48	0.07	1.26
R234A	5.21	0.08	1.20
R235A	4.41	0.03	1.02
R189A/R193A	5.03	0.07	1.16
R189A/R234A	4.72	0.03	1.09
R189A/R235A	4.20	0.05	0.97
R193A/R235A	4.51	0.03	1.04
R234A/R235A	4.30	0.05	0.99
R189A/R193A/R234A	4.59	0.01	1.06
R189A/R193A/R235A	4.37	0.14	1.01
R189A/R234A/R235A	4.42	0.08	1.02
R193A/R234A/R235A	4.49	0.06	1.03
R189A/R193A/R234A/R235A	4.43	0.05	1.02

Group 2	Average	SD	Fold Change
VC	4.22	0.06	1.00
WT	5.71	0.06	1.35
R191A	4.75	0.03	1.13
R196A	4.52	0.06	1.07
R197A	4.40	0.04	1.04
R235A	4.07	0.12	0.97
R191A/R196A	4.26	0.03	1.01
R191A/R235A	4.04	0.09	0.96
R196A/R235A	4.18	0.18	0.99

Figure S1. Doubling time of RTA mutants. Yeast cells were transformed with each RTA mutant. Cells from 3 different colonies were grown in glucose supplemented medium for at least 48 hours. Optical density was measured at 600 nm every 30min. Doubling time (hours) calculated from three independent growth curves for each mutant and standard deviation (SD) are shown.

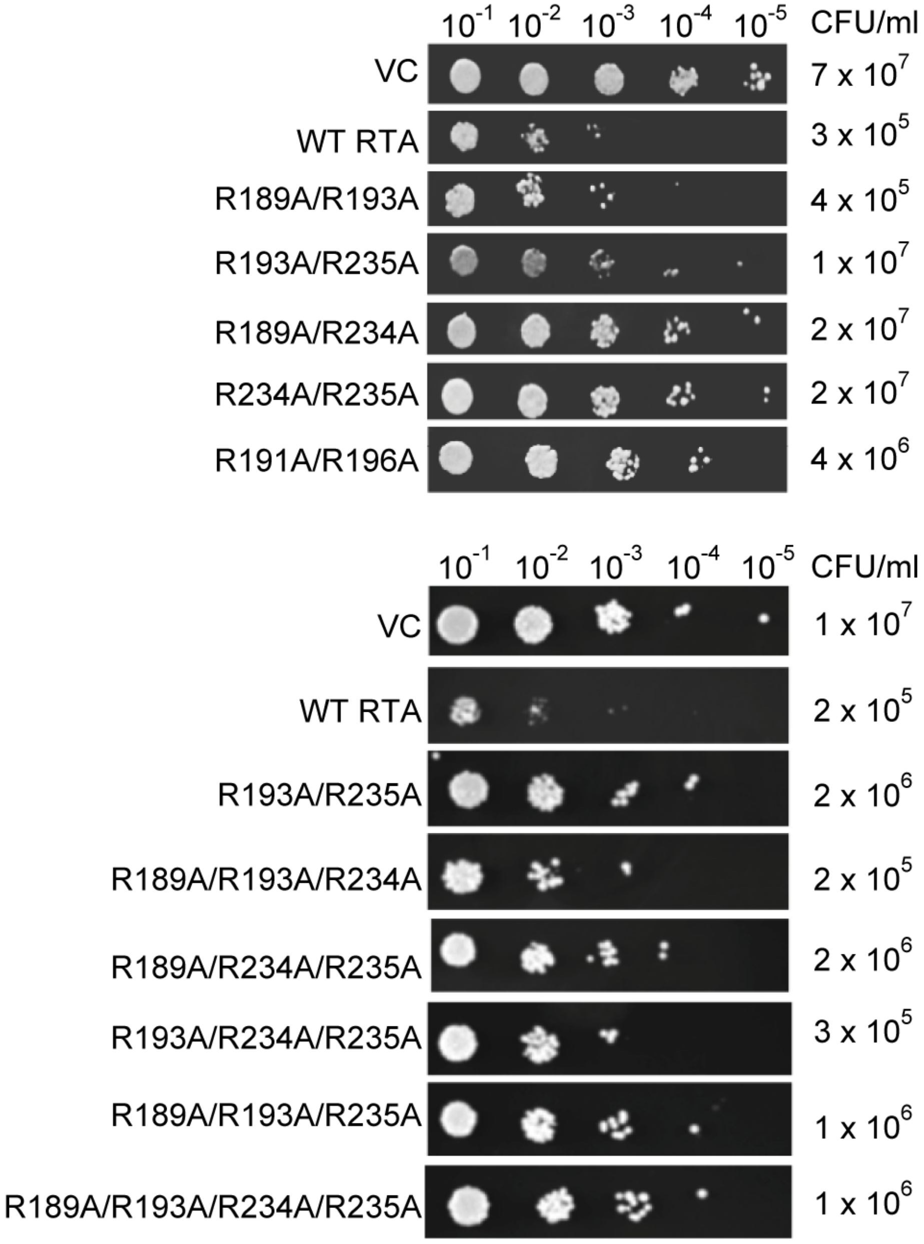

Figure S2. Viability of double and triple arginine mutants. Yeast cells were transformed with either WT or mutant RTA and spotted on glucose containing medium at 8 hours post induction.

+ Majority

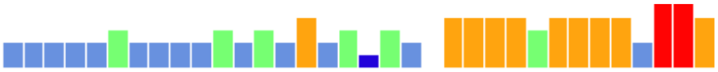

```
Majority                        A A A G A A A A A A E K K E E E K E E E - E E S D D D M G F S L F D - -
                                     290                 300                 310                 320

Saccharomyces cerevisiae P0     A A S G - - - D A A P A E E A A A E E - E E E S D D D M G F G L F D          312
Saccharomyces cerevisiae P1     G E A G E A E A - - - E K E E E E A K E - - E S D D D M G F G L F D          106
Saccharomyces cerevisiae P2     A G G D A A - - - - E E E K E E - - E A K E E S D D D M G F G L F D          110
Schizosaccharomyces pombe P0    A G G E - - - A E A P A A E A A A E E - E E E S D E D M G F G L F D          312
Schizosaccharomyces pombe P1    P A A A D G E A P A E E K E E A K E E E - - E S D E D M G F G L F D          109
Schizosaccharomyces pombe P2    A A G G A A P A A E E A A K E E A K E E - E E S D E D M G F G L F D          110
Ricinus communis P0             V A A A G A P A A A A K E E E K K E E P A E E S D D D M G F S L F D          320
Ricinus communis P1             G A A A A A P A V E E K K E E P K - - - - E E S D D D M G F S L F D          110
Ricinus communis P2             A A G G A A A P A A E A K K E E K V E E K E E S D D D M G F S L F D          113
Ricinus communis P3             A A A A A E A P A E E K K K E E P A - - - E E S D D D M G F S L F D          122
Arabidopsis thaliana P0         A D A G G G A P A A A K V E E K E E S D E E D Y G G D - - F G L F D E E      287
Arabidopsis thaliana P1         G A A A A P A A E E K K K D E P A - - - - E E S D G D L G F G L F D          112
Arabidopsis thaliana P2         G G G G G G A P A A E S K K E E K K E E K E E S D D D M G F S L F E          115
Zea mays P0                     A G D S G A S A A - P K E E E K A A E P E E E S D E E M G F S L F D D        319
Zea mays P1                     A A A A A A P A V E E K K E E A K - - - - E E S D D D M G F S L F D          109
Zea mays P2                     A A G G - R A P A A E A K K E E K V E E K E E S D D D M G F S L F D          112
Zea mays P3                     G A A A - E A P K E E K K E E E K - - - - E E S D D D M G F S L F D          120
Oryza sativa P0                 A D S G A A A P S A A K E E E K K E E P E E E S D G D L G M S L F D          319
Oryza sativa P1                 G G A A A A P A A E E K K E E A K - - - - E E S D D D M G F S L F D          110
Oryza sativa P2                 A A G G G A A P Q S E A K K E E K V E E K E E S D D D M G F S L F D          113
Triticum Aestivum P0            A D S G A A A P S A A K E E E K K E E P E E E S D G D L G M S L F D          319
Triticum Aestivum P1            G A A A A E E K K E E K K E E A K - - - - E E S D D D M G F S L F D          110
Triticum Aestivum P2            A A G G A A A P A A E S K K E E K V V E K E E S D D D M G F S L F D          113
Hordeum vulgare P0              A A S G G A A A A A P K E E E K K E E P E E E S D G E M G F S L F D D        320
Hordeum vulgare P1              G A A V E E D K K E E E K E E E K E E - - - - S D E D M G F G L F D          110
Hordeum vulgare P2              S A G G A A A P A A E S K K E E K V V E K E E S D D D M G F S L F D          113
Mus musculus P0                 T T A A P A A A A A P A K A E A K E E - S E E S D E D M G F G L F D          317
Mus musculus P1                 A P S T A A A P A E E K K V E A K K E E S E E S E D D M G F G L F D          114
Mus musculus P2                 P A A G S A P A A A E E K K D E K K E E S E E S D D D M G F G L F D          115
Homo sapiens P0                 T T A A P A A A A A P A K V E A K E E - S E E S D E D M G F G L F D          317
Homo sapiens P1                 A P S T A A A P A E E K K V E A K K E E S E E S D D D M G F G L F D          114
Homo sapiens P2                 P A A G S A P A A A E E K K D E K K E E S E E S D D D M G F G L F D          115
```

Figure S3. Alignment of C-terminal sequences of stalk P-proteins from different species. C-terminal sequences of yeast (*Saccharomyces cerevisiae*, P0-NP_013444.1, P1-NP_010202.1, P2-NP_010670.3; *Schizosaccharomyces pombe*, P0-NP_588393.1, P1-NP_593883.1, P2-NP_596513.1), castor bean (*Ricinus communis*, P0-XP_002526873.1, P1-XP_015578010, P2- EEF33307.1), *Arabidopsis* (*Arabidopsis thaliana*, P0-NP_001078125.1, P1- NCBI Accession NP_849569.1, P2-NP_180340.1), maize (*Zea mays*, P0-NP_001105482.1, P1-NP_001105701.1, P2-NP_001105377.1, P3-NP_001105389.1), rice (*Oryza sativa*, P0-XP_015650041.1, P1-XP_015650060.1, P2-XP_015623698.1), wheat (*Triticum aestivum*, P0-3J61_q, P1-3J61_u, P2-3J61_s), barley (*Hordeum vulgare*, P0 -BAJ92405.1, P1- CCU78173.1, P2-BAJ92129.1), mouse (*Mus musculus*, P0-NP_031501.1, P1-NP_061341.1, P2-NP_080296.3) and human (*Homo sapiens*, P0-NP_000993.1, P1- NP_000994.1, P2- NP_000995.1) P-proteins were aligned using MegAlign, Lasergene (DNASTAR, Inc. Madison, WI, USA).

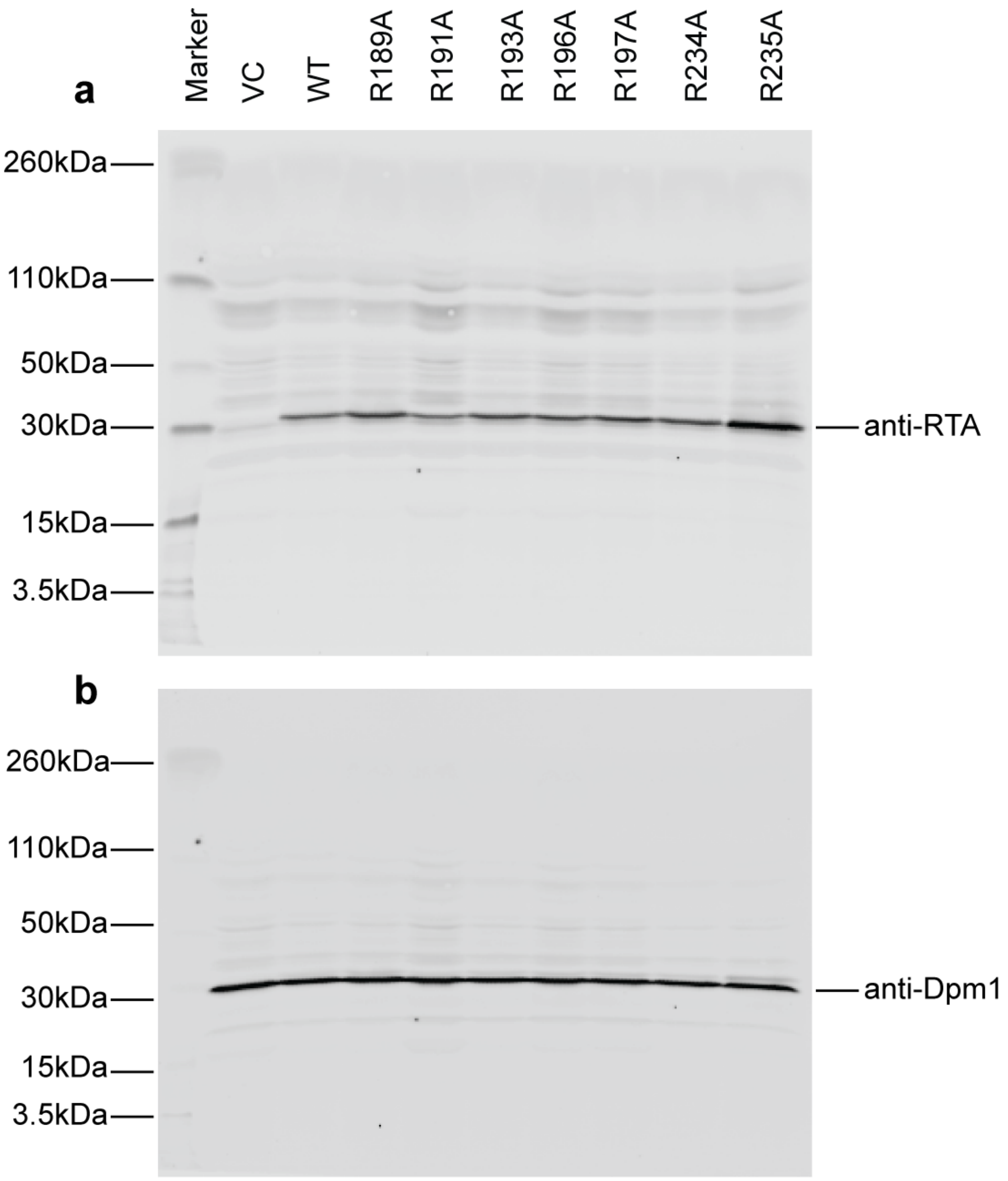

Figure S4. Full-length image of Fig. 1e. Yeast cells were transformed with VC, WT RTA and single RTA mutants. Expression of the toxin was induced for 4 hours. **(a)** Total protein from 1 OD cells was loaded on a 15% SDS-PAGE gel, transferred to nitrocellulose and probed with monoclonal antibody against RTA. **(b)** The same blot was stripped with 8M guanidine hydrochloride and re-probed with anti-dolichol phosphate mannose synthase (Dpm1) antibody.

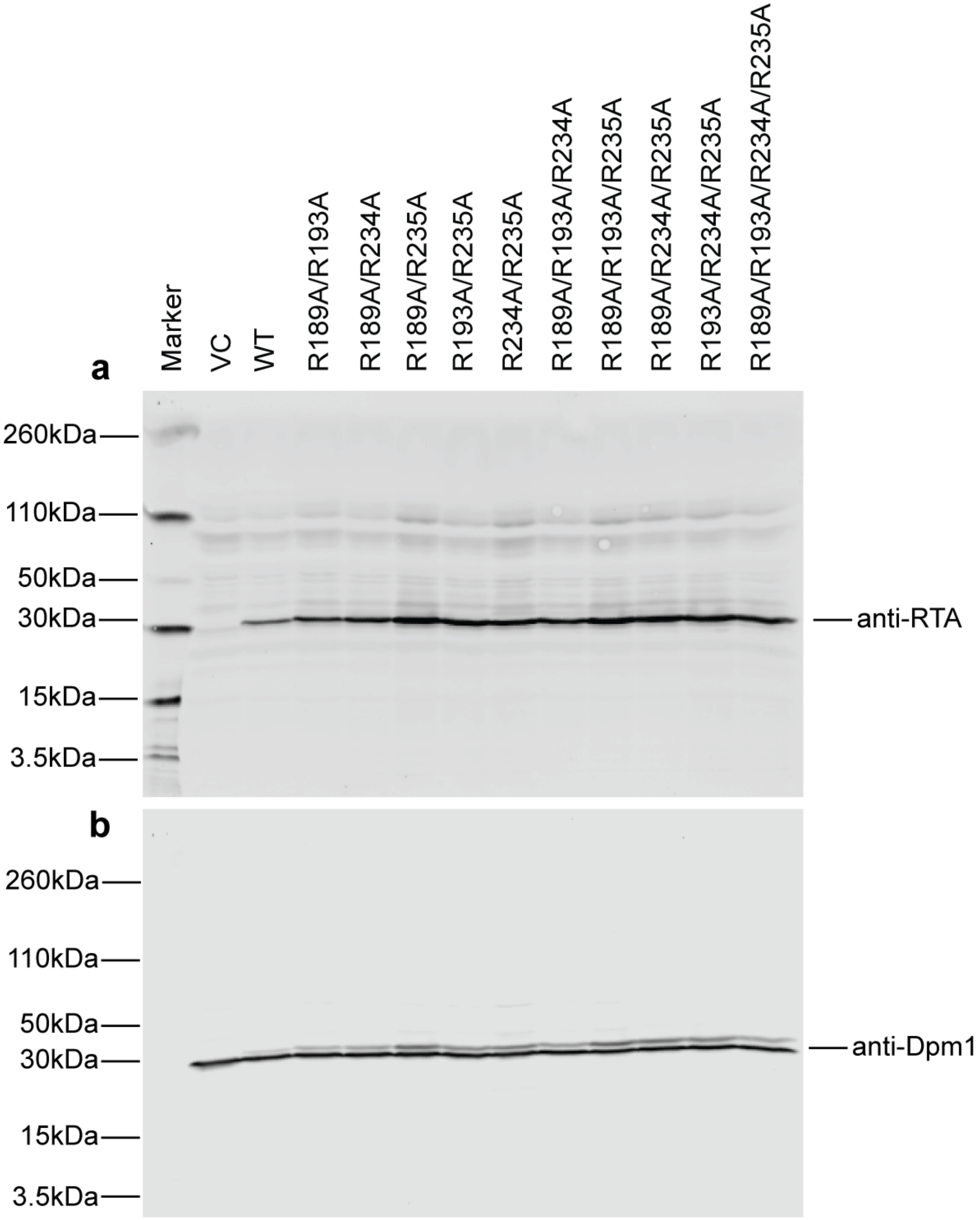

Figure S5. Full-length image of Fig. 4b. Yeast cells were transformed with VC, WT RTA, double, triple and quadruple RTA mutants. Expression of the toxin was induced for 4 hours. **(a)** Total protein from 1 OD cells was loaded on a 15% SDS-PAGE gel, transferred to nitrocellulose and probed with monoclonal antibody against RTA. **(b)** The same blot was stripped with 8M guanidine hydrochloride and re-probed with anti-dolichol phosphate mannose synthase (Dpm1) antibody.
